# The Case Report of *Klebsiella quasipneumoniae* subsp. *similipneumoniae* ST138-K1/O5 in a Clinically Vulnerable Patient

**DOI:** 10.3390/microorganisms14102357

**Published:** 2026-10-15

**Authors:** Santiago Munilla, Romina Papa-Ezdra, Nicolás F. Cordeiro, Inés Bado, Verónica Seija, Rafael Vignoli, Nilton Lincopan, Rosario Palacio, Virginia Garcia-Fulgueiras

**Affiliations:** 1 Área Microbiología, Departamento de Laboratorio Clínico, Hospital de Clínicas Dr. Manuel Quintela, Facultad de Medicina, Universidad de la República, Montevideo CP 11600, Uruguay; santiago7210@gmail.com (S.M.); vseija1@gmail.com (V.S.); 2 Departamento de Bacteriología y Virología, Instituto de Higiene, Facultad de Medicina, Universidad de la República, Montevideo CP 11600, Uruguay; rpapa@higiene.edu.uy (R.P.-E.); ncordeiro@higiene.edu.uy (N.F.C.); ibado@higiene.edu.uy (I.B.); rvignoli@higiene.edu.uy (R.V.); 3 Department of Microbiology, Institute of Biomedical Sciences, University of São Paulo, São Paulo 05508-000, Brazil; lincopan@usp.br; 4 One Health Brazilian Resistance Project (OneBR), São Paulo 05508-000, Brazil; 5 Antimicrobial Resistance Institute of São Paulo (ARIES), São Paulo 01301-100, Brazil

**Keywords:** bacteremia, *Klebsiella quasipneumoniae* subsp. *similipneumoniae*, ST138, K1/O5 locus

## Abstract

Infections caused by the *Klebsiella pneumoniae* species complex (KpSC) are particularly challenging to manage, especially in healthcare systems. Members of the KpSC are frequently identified as *K. pneumoniae* by conventional automated or proteomic systems. The aim of this study was to characterize a KpSC isolate obtained from a clinically vulnerable patient and to describe the patient’s clinical features. The patient’s clinical data were obtained from the medical history. Identification and antibiotic susceptibility testing were performed using different approaches. Whole Genome Sequencing (WGS) was conducted, and in silico analysis using available databases was undertaken to predict antibiotic resistance determinants, plasmid incompatibility groups, sequence type (ST), capsular K-type, O-antigen, and virulence genes. A *K. pneumoniae* isolate (K87) was recovered from blood cultures and characterized as *K. quasipneumoniae* subsp. *similipneumoniae* by WGS. K87 belongs to ST138 and carries the predicted K1/O5 locus and the *bla*_OKP-B-2_ gene. The virulome included type III fimbriae, ferric iron uptake systems, and metabolic functions. After months of hospitalization, the patient showed clinical deterioration and died. This is the first report of bacteremia caused by *Klebsiella quasipneumoniae* subsp. *similipneumoniae* ST138-K1/O5 in a clinically vulnerable patient in Uruguay (South America).

## 1. Introduction

*Klebsiella pneumoniae* (*K. pneumoniae*), a major and prevalent opportunistic microorganism, is one of the priority pathogens included in the ESKAPE group [1,2,3]. It causes a broad spectrum of clinical manifestations, including pneumonia, meningitis, pyogenic liver abscesses, urinary tract infections, bacteremia, and sepsis [2]. In bloodstream infections (BSIs), *K. pneumoniae* presents a high prevalence, particularly in elderly patients with comorbidities [4]. It is a member of the *K. pneumoniae* species complex (KpSC) that is composed of seven closely related species and subspecies subclassified into phylogroups (Kp) [1]. KpSC isolates are often identified as *K. pneumoniae* by automated systems or the matrix-assisted laser desorption ionization time-of-flight mass spectrometry (MALDI-TOF MS) [5]. By using a whole-genome sequencing (WGS) approach, it is possible to identify all members at the level of species and subspecies (subsp.). In this regard, KpSC are characterized by: *K. pneumoniae* (Kp1), *K. quasipneumoniae* subsp. *quasipneumoniae* (Kp2), *K. quasipneumoniae* subsp. *similipneumoniae* (Kp4), *K. variicola* subsp. *variicola* (Kp3), *K. variicola* subsp. *tropica* (Kp5), *K. quasivariicola* (Kp6) and *K. africana* (Kp7) [1,5].

The first description of *K. quasipneumoniae* subsp. *similipneumoniae* (Kqs) was from human blood cultures in 2014 [6]. Since then, there have been few reports of human invasive infections caused by this microorganism [7,8].

BSIs are an important cause of morbidity and mortality around the world, affecting millions of individuals annually [9] and frequently leading to poor clinical outcomes or death [10]. Patient mortality is multifactorial, driven by host-related factors (age, presence of comorbidities, site of infection acquisition, focus of bacteremia, severity of initial presentation, antibiotic treatment received), as well as pathogen-related characteristics (virulence factors, among others) [11].

To characterize the different KpSC members from invasive infections, sequence types (STs) must be studied, since many of them are associated with the pathogenicity of individual strains [1,12]. Different STs have been described in Kqs strains from human invasive infections [7,13], but there are few reports of ST138 in clinical settings, and those available do not involve BSIs [14,15].

Virulence factors play a critical role in the development of clinical infections [4]. Some of these factors are present in all isolates, such as surface polysaccharides (capsular K-type and lipopolysaccharide O-antigens) or adhesion type III fimbriae; however, others are related to a specific hypervirulent phenotype [1]. K1-type is particularly significant for its anti-phagocytic role and resistance to serum killing [16]. Siderophores for iron uptake are also frequently present in pathogenic isolates [17,18]. K1-type, genes for type I and type III fimbriae, type IV pili, siderophores, and allantoin utilization (in some cases) were the most prevalent virulence factors identified in Kqs isolates from clinical infections [14,15,19,20].

In this work, we report a *Klebsiella quasipneumoniae* subsp. *similipneumoniae* ST138-K1/O5 isolate in a clinically vulnerable patient.

## 2. Materials and Methods

### Case Presentation

The patient was admitted to “Hospital de Clínicas Dr. Manuel Quintela” (2019), presenting with abdominal distension and pleural effusion. The patient’s medical history included type 2 diabetes mellitus and severe liver cirrhosis secondary to non-alcoholic steatohepatitis. The Child-Pugh score was used to classify the severity of liver disease. To assess the Child-Pugh score, five parameters are considered: total serum bilirubin, serum albumin, prothrombin time, presence and severity of ascites, and degree of hepatic encephalopathy. Each parameter is assigned a score [21]. The patient’s clinical data were collected by review of the medical records. The study protocol was approved by the Medical Ethics Committee of “Hospital de Clínicas Dr. Manuel Quintela” (6 June 2018). The Hospital Ethics Committee reviews each protocol and assigns an approval date. The Committee does not provide an identification or reference number; therefore, the approval date constitutes the formal resolution of the Hospital Ethics Committee.

During hospitalization, the patient developed bacteremia. The Pitt bacteremia score was calculated based on five clinical parameters: body temperature, hypotension, mechanical ventilation, cardiac arrest, and mental status. Each parameter was assigned a specific number of points according to severity [22]. Two sets of blood cultures were collected and incubated in the BacT/ALERT^®^ 3D system (bioMérieux, Marcy l’Étoile, France). Microorganism identification was performed using the VITEK^®^-2 system (bioMérieux, Marcy l’Étoile, France) and MALDI-TOF MS (Bruker, Billerica, MA, USA). Antimicrobial susceptibility testing was performed using the automated system, and results were interpreted according to the Clinical and Laboratory Standards Institute (CLSI) guidelines [23]. The same pathogen was identified in both blood cultures.

Genomic DNA extraction was carried out using the NZY microbial gDNA isolation kit, according to the manufacturer’s instructions (NZYTech Genes & Enzymes, Lisbon, Portugal), followed by WGS. The quality of the extracted DNA samples was measured using a NanoDrop 1000 spectrophotometer (Thermo Fisher, Wilmington, DE, USA). DNA quantification was carried out using a Qubit 3.0 fluorometer and the dsDNA HS Assay kit (Thermo Fisher Scientific, Waltham, MA, USA). Illumina libraries were prepared using the Nextera XT DNA Library Prep Kit along with the Nextera XT Index kit (Illumina Inc., San Diego, CA, USA) and sequenced with an Illumina MiniSeq device and a MiniSeq High Output reagent kit (Illumina Inc., San Diego, CA, USA), following a 2 × 151 bp paired-end approach. Short reads were quality-checked and trimmed using TrimGalore v0.6.10 (https://github.com/FelixKrueger/TrimGalore, Babraham Institute, Cambridge, UK, accessed on 30 August 2025), and genome assembly was performed using Unicycler v0.5.1 (https://github.com/rrwick/Unicycler, University of Melbourne, Melbourne, Australia, accessed on 30 August 2025).

Taxonomy assignment was performed using average nucleotide identity (ANI) against the genome taxonomy database v0.3.0 (https://gtdb.ecogenomic.org/, FastANI v1.32, ANI ≥ 95%, Australian Centre for Ecogenomics, The University of Queensland, Brisbane, Australia, accessed on 30 August 2025). Other parameters included N50 and assembly coverage (NCBI, 30 August 2025). Capsular K-type and O-antigen (O) locus were determined using Kaptive v3.1.0 (https://github.com/klebgenomics/Kaptive, Tom Stanton, Monash University, Melbourne, Australia, accessed on 30 August 2025). ST was investigated using the Pathogenwatch platform (https://pathogen.watch/, Center for Genomic Pathogen Surveillance, Wellcome Sanger Institute, Hinxton, UK, accessed on 30 August 2025). The virulence score was determined using the same platform. Virulence genes were predicted on the Institut Pasteur website (https://bigsdb.pasteur.fr/klebsiella/, Institut Pasteur, Paris, France, accessed on 1 September 2025). The occurrence of antimicrobial resistance genes was predicted using the ResFinder 4.1 online service (minimum identity 80%, minimum coverage 60%, Technical University of Denmark, Kongens Lyngby, Denmark, 1 September 2025) [24]. On the other hand, the presence of putative plasmid incompatibility groups was predicted using the PlasmidFinder 2.1 suite (minimum identity 95%, minimum coverage 60%, Technical University of Denmark, Kongens Lyngby, Denmark, 1 September 2025), available on the Center for Genomic Epidemiology website (https://www.genomicepidemiology.org, accessed on 1 September 2025).

A string test procedure was performed to determine whether the strain was hypermucoviscous. A positive string test is defined as the formation of a viscous string > 5 mm in length when a bacterial colony grown on a blood agar plate is stretched with an inoculation loop [25].

## 3. Results

### 3.1. Patient

A 60-year-old female was admitted to the hospital presenting with abdominal distension and pleural effusion. The patient’s medical history included type 2 diabetes mellitus and severe liver cirrhosis secondary to non-alcoholic steatohepatitis. Child-Pugh class C (score 10) indicates severe liver disease. The patient presented with a history of recurrent hepatic encephalopathy, ascites secondary to portal hypertension, persistent hypoalbuminemia, and required multiple hospitalizations.

In the last hospitalization, after 116 days, the patient showed clinical deterioration and experienced a febrile episode accompanied by chills (fever up to 38 °C), with a prognostic severity measure of Pitt bacteremia score > 2, indicating severe BSI. Prior to the onset of bacteremia, empirical treatment with vancomycin and meropenem was administered (for 10 days). The antibiotic treatment was completed 20 days before the onset of bacteremia, and the patient received no further antibiotic treatment thereafter. The episode of bacteremia was classified as a primary, hospital-acquired bloodstream infection of unknown origin. The patient had a central venous catheter and a pleural drain; no urinary catheter was in place. However, neither device was considered a clinically suspected source, and no associated positive cultures were obtained.

The laboratory findings included thrombocytopenia, 47 × 10^3^/mm^3^; a decline in leukocyte count to 1.78 × 10^3^/mm^3^ on the day of onset of the bacteremic episode (subsequently recovering to 8.88 × 10^3^/mm^3^ after day 5); elevated serum creatinine, 2.33 mg/dL (already present at baseline); hyperbilirubinemia, 2.34 mg/dL; erythrocyte sedimentation rate of 30 mm/h; and C-reactive protein level of 52.6 mg/L. Upon clinical suspicion of bacteremia and immediately after blood culture collection, empirical antimicrobial therapy was initiated with meropenem 500 mg intravenously every 8 h, administered as a 5 h extended infusion, with the dose adjusted according to the patient’s renal function (creatinine clearance, 42.6 mL/min).

Two sets of blood cultures were obtained. In the first set, the aerobic bottle became positive at 11.06 h, with an inoculated blood volume of 8.1 mL; in the second set, positivity was reached at 10.34 h, with an inoculated blood volume of 9.2 mL. The concordance in microbiological identification and antimicrobial susceptibility profiles between the two sets (as discussed below), together with the time to positivity and adequate inoculated blood volumes, supported the classification of the finding as true bacteremia. No follow-up blood cultures were obtained after the initiation of antimicrobial therapy.

The absence of clinical improvement, the unknown source of infection, the pharmacokinetic alterations secondary to hepatic disease, and the context of prolonged hospitalization led to the decision not to de-escalate therapy despite the susceptibility of the isolate. The regimen was maintained for 14 days, until the patient’s death.

It should be considered that, in this case, the patient presented with bacteremia and had pre-existing severe liver disease.

### 3.2. Bacterial Identification and Antimicrobial Susceptibility Assays

A *K. pneumoniae* isolate (named K87 in our laboratory) grew in both blood culture samples and displayed susceptibility to ampicillin/sulbactam, piperacillin/tazobactam, cefotaxime, ceftazidime, cefepime, ertapenem, meropenem, imipenem, amikacin, gentamicin, ciprofloxacin, and trimethoprim-sulfamethoxazole. K87 was resistant to ampicillin (Table 1).

### 3.3. Whole-Genome Sequencing

To explore the genetic features of K87, a draft genome was determined through a WGS approach. Assembly statistics resulted in a draft genome length of 5.34 Mb, 74 contigs, assembly coverage of 91.61%, ANI of 99.28%, N50 of 357 kb (NCBI, 30 August 2025) and GC content of 58% (GenBank assembly: GCA_052373715.1, BioProject ID: PRJNA1303087, BioSample ID: SAMN50494446, 7 August 2025).

K87 was characterized as *K. quasipneumoniae* subsp. *similipneumoniae* (RefSeq *Klebsiella*_*quasipneumoniae*_subsp_*similipneumoniae*/0026.fna.gz, Pathogenwatch, 30 August 2025).

### 3.4. Isolate Characterization

**Figure 1:**
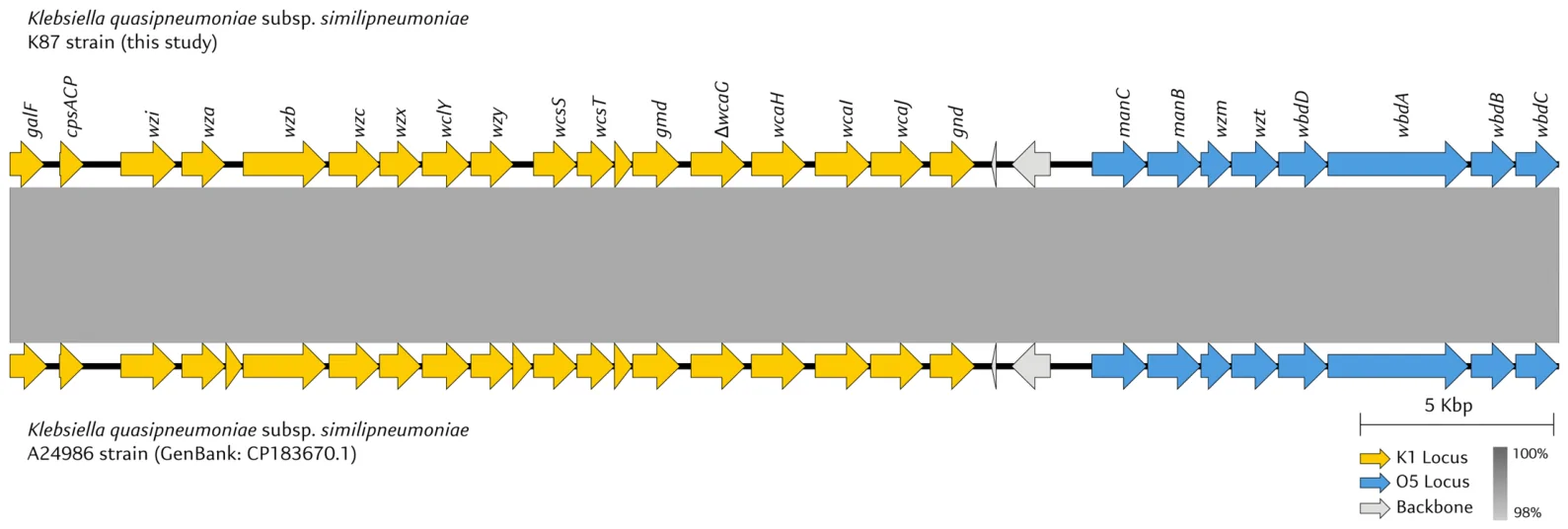
Schematic representation of the K1 locus and O5 locus identified in Kqs K87 (this study) in comparison to the A24986 reference strain (GenBank accession No. CP183670.1). Arrows represent coding sequences (CDS) labeled with gene names and displayed according to gene orientation; the gray shades indicate regions of homology.

K87 belonged to ST138 and carried the predicted K1 and O5 loci. The capsular K1-type and O5 locus (Figure 1) were identical to those of *K. quasipneumoniae* subsp. *similipneumoniae* (A24986 strain, GenBank accession No. CP183670.1).

The *bla*_OKP-B-2_ antimicrobial resistance gene was identified; this gene is an intrinsic, chromosomally encoded β-lactamase typical of the KpSC phylogroup. The *bla*_OKP-B-2_ encodes a β-lactamase that confers resistance to ampicillin, amoxicillin, carbenicillin, and ticarcillin.

PlasmidFinder establishes the presence of an IncFIB(K) replicon sequence but does not determine the structure of a complete plasmid or its association with the different genes.

The virulome included type III fimbriae (*mrkABCDFHIJ*), the siderophore enterobactin (*entABCDEF*), the ferric iron uptake system (*kfuABC*), and different metabolic functions for allantoin (*allABCDRS*), a two-component system characterized as a redox-responsive regulator activated under anaerobic/microaerobic conditions (*arcAB*), acyl-CoA synthetase (*fdrA*), glycerate 3-kinase (*glxAKR*), glyoxylate carboligase (*gcl*), and hydroxypyruvate isomerase (*hyi*).

Genes for the hypermucoid phenotype (*rmpADC* and *rmpA2*), yersiniabactin (*ybST*), colibactin (*cbST*), aerobactin (*abST*), and salmochelin (*smST*) were not detected, and the virulence score was 0. Isolate K87 was not a hypermucoviscous strain by the string test method.

## 4. Discussion

We presented a report of bacteremia in a patient with severe liver cirrhosis secondary to non-alcoholic steatohepatitis. Prior to the episode of bacteremia, the patient was exposed to vancomycin and meropenem. This therapy could have contributed to antibiotic-driven bacterial selection or microbiota disruption, potentially predisposing the patient to the subsequent infection.

Regarding the BSI isolate, automated systems and MALDI-TOF MS cannot reliably distinguish species and subsp. within the KpSC, meaning that isolates are routinely identified as *K. pneumoniae*. Kqs was identified for the first time in 2014 using a WGS approach [6]. Although Kqs isolates have been documented in diverse clinical samples across age groups [7,8,26,27,28], here we focus on blood culture isolates [7,29]. Cases of BSIs caused by Kqs from capsular K1-type remain scarce, with identified isolates belonging to STs other than ST138 [8,13,30].

In public databases, clinical KpSC ST138 isolates have been reported exclusively in Kqs. These ST138-K1 strains were isolated from peritoneal fluid and surgical site infections [14,15]. The former harbored genes encoding type I and III fimbriae, type IV pili, and siderophores [14]. The latter exhibited virulence factors involved in type I and III fimbriae, allantoin metabolism, and iron acquisition [15]. In our country, genes encoding type III fimbriae and ferric iron uptake systems were previously identified in *K. pneumoniae* blood culture isolates from pediatric patients. However, the identification was performed by automated systems, and it remains unknown whether other members of the KpSC were also present [2]. Although the K87 isolate achieved a virulence score of 0, it was possible to identify certain virulence factors associated with metabolic functions. In general, these factors were poorly characterized or completely absent in Kqs isolates. Specifically, systems related to allantoin utilization, anaerobic and microaerobic metabolism, acyl-CoA synthetase, glycerate 3-kinase, glyoxylate carboligase, and hydroxypyruvate isomerase were rarely detected [15].

These findings, which have rarely been reported in other Kqs strains to date, may contribute to the characterization of uncommon ST138 isolates.

The O5 lipopolysaccharide antigen had previously been reported in *K. pneumoniae* blood culture isolates as one of the most frequent serotypes in BSIs [31,32]. However, based on data available to date, Kqs ST138-K1/O5 isolates have not been reported in association with bacteremia.

The only antimicrobial resistance gene identified in the K87 isolate was *bla*_OKP-B-2_. This gene encodes an intrinsic, chromosomally encoded β-lactamase that confers resistance only to ampicillin, amoxicillin, carbenicillin, and ticarcillin. In this isolate, we could not identify any acquired antimicrobial resistance mechanisms.

The phenotypic and genomic characteristics of the isolate suggest an opportunistic invasive infection in a highly vulnerable host. In this context, based on the patient’s clinical history, the cause of death was considered to be multifactorial. Bacteremia may have contributed to the patient’s clinical deterioration and accelerated the progression toward death. However, it was not possible to establish whether the bacterial infection was the cause of hepatic failure.

We would like to emphasize that this study had some limitations. The short-read assembly precluded confident plasmid replicon typing and determination of the chromosomal or plasmid localization of genes, as well as assessment of virulence gene completeness.

## 5. Conclusions

In this work, using a WGS approach, we identified a Kqs ST138-K1/O5 isolate from bacteremia in a clinically vulnerable patient with severe liver cirrhosis.

Kqs isolate harbored virulence genes related to adhesion, iron acquisition, and metabolic pathways. Some metabolic pathways were rarely detected, and our results are a contribution to the scientific community.

Surveillance using WGS is necessary for the accurate detection and identification of KpSC pathogens in hospital settings.

To the best of our knowledge, this report represents the first documented case of bacteremia caused by *Klebsiella quasipneumoniae* subsp. *similipneumoniae* ST138-K1/O5 in a patient with decompensated cirrhosis in Uruguay (South America).

## Figures and Tables

**Table 1:** Susceptibility profile of the K87 isolate.

Antibiotic	MIC (mg/L)	Interpretation
Ampicillin	≥32	Resistant
Ampicillin/sulbactam	≤4	Susceptible
Piperacillin/tazobactam	≤4	Susceptible
Cefotaxime	≤1	Susceptible
Ceftazidime	≤1	Susceptible
Cefepime	≤1	Susceptible
Ertapenem	≤0.5	Susceptible
Meropenem	≤0.25	Susceptible
Imipenem	≤0.25	Susceptible
Amikacin	≤2	Susceptible
Gentamicin	≤1	Susceptible
Ciprofloxacin	≤0.25	Susceptible
Trimethoprim-sulfamethoxazole	≤20	Susceptible

MIC, minimum inhibitory concentration.

## Data Availability

Whole-genome sequence data were deposited in the GenBank database under BioProject acc. No. PRJNA1303087.

## References

[1] Wyres K.L., Lam M.M.C., Holt K.E. (2020). Population Genomics of *Klebsiella pneumoniae*. Nat. Rev. Microbiol..

[2] Araújo L., Papa-Ezdra R., Ávila P., Iribarnegaray V., Bado I., Telechea H., Garcia-Fulgueiras V., Vignoli R. (2024). Great Plasticity in a Great Pathogen: Capsular Types, Virulence Factors and Biofilm Formation in ESBL-Producing *Klebsiella pneumoniae* from Pediatric Infections in Uruguay. Antibiotics.

[3] Marzouk E., Abalkhail A., ALqahtani J., Alsowat K., Alanazi M., Alzaben F., Alnasser A., Alasmari A., Rawway M., Draz A. (2024). Proteome Analysis, Genetic Characterization, and Antibiotic Resistance Patterns of *Klebsiella pneumoniae* Clinical Isolates. AMB Express.

[4] Fostervold A., Raffelsberger N., Hetland M.A.K., Bakksjø R., Bernhoff E., Samuelsen Ø., Sundsfjord A., Afset J.E., Berntsen C.F., Bævre-Jensen R. (2024). Risk of Death in *Klebsiella pneumoniae* Bloodstream Infections Is Associated with Specific Phylogenetic Lineages. J. Infect..

[5] Lam M.M.C., Wick R.R., Watts S.C., Cerdeira L.T., Wyres K.L., Holt K.E. (2021). A Genomic Surveillance Framework and Genotyping Tool for *Klebsiella pneumoniae* and Its Related Species Complex. Nat. Commun..

[6] Brisse S., Passet V., Grimont P.A.D. (2014). Description of *Klebsiella quasipneumoniae* sp. Nov., Isolated from Human Infections, With Two Subspecies, *Klebsiella quasipneumoniae* subsp. *quasipneumoniae* subsp. Nov. and *Klebsiella quasipneumoniae* subsp. *similipneumoniae* subsp. Nov., And Demonstration That *Klebsiella singaporensis* Is a Junior Heterotypic Synonym of *Klebsiella variicola*. Int. J. Syst. Evol. Microbiol..

[7] Ding J., Xu M., Xia Y., Kang X., Yu Y., Chang J., Hu Z., He P., Yao Y., Shen N. (2025). Genomic and Phenotypic Characterization of Extensively Drug-Resistant Clinical *Klebsiella quasipneumoniae* subsp. *similipneumoniae* ST1929 Isolates. Infect. Drug Resist..

[8] Saito S., Nakashima S., Endo K., Narita K., Hatakeyama Y., Suzuki Y., Nakano R., Suzuki Y., Yano H., Fujiwara T. (2026). Invasive Infection Caused by a Hypervirulent ST367-KL1 *Klebsiella quasipneumoniae* subsp. *similipneumoniae*: First Case Report in Japan. Jpn. J. Infect. Dis..

[9] Lin M.F., Sun B., Liu Z.Y., Tang P., Zhang L.J., Wang Y.Y. (2020). Evaluation of the Clinical Diagnostic Value of Traditional Inflammatory Markers and Novel Biomarkers in Intracellular Bacterial Bloodstream Infections. Cytokine.

[10] Van Walraven C., Wong J. (2014). Independent Influence of Negative Blood Cultures and Bloodstream Infections on In-Hospital Mortality. BMC Infect. Dis..

[11] Schwab F., Geffers C., Behnke M., Gastmeier P. (2018). ICU Mortality Following ICU-Acquired Primary Bloodstream Infections According to the Type of Pathogen: A Prospective Cohort Study in 937 Germany ICUs (2006-2015). PLoS ONE.

[12] Bahati S.Y., Maghembe R.S. (2025). Pangenome Analysis of Tanzanian Clinical *Klebsiella pneumoniae* Reveals Pandemic Clones with High Genome Plasticity and Versatile Mobilome, Virulome, and Resistome Profiles. Microbiol. Spectr..

[13] Okomo U., Senghore M., Darboe S., Bojang E., Zaman S.M.A., Hossain M.J., Nwakanma D., Le Doare K., Holt K.E., Hos N.J. (2020). Investigation of Sequential Outbreaks of *Burkholderia cepacia* and Multidrug-Resistant Extended Spectrum β-Lactamase Producing *Klebsiella* species in a West African Tertiary Hospital Neonatal Unit: A Retrospective Genomic Analysis. Lancet Microbe.

[14] Lobato A.R.F., Souza M.J.S., Pereira E.S., Cazuza T.B., Silva A., Baraúna R.A., Brasiliense D.M. (2025). Emergence of NDM-7-Producing *Klebsiella quasipneumoniae* subsp. *similipneumoniae* ST138 in a Hospital from the Northern Region of Brazil. Microorganisms.

[15] do Valle Barroso M., Oshiro B.d.S., Nogueira M.C.L., Casella T. (2025). Genomic Insights into a Colonization-Infection Progression by a Carbapenemase-Producing *Klebsiella quasipneumoniae* K1 Serotype. J. Glob. Antimicrob. Resist..

[16] Wang T.C., Lin J.C., Chang J.C., Hiaso Y.W., Wang C.H., Chiu S.-K., Fung C.P., Chang F.Y., Siu L.K. (2021). Virulence among Different Types of Hypervirulent *Klebsiella pneumoniae* with Multi-Locus Sequence Type (MLST)-11, Serotype K1 or K2 Strains. Gut Pathog..

[17] Russo T.A., Marr C.M. (2019). Hypervirulent *Klebsiella pneumoniae*. Clin. Microbiol. Rev..

[18] Hyun M., Lee J.Y., Kim H.A. (2024). Clinical and Microbiologic Analysis of *Klebsiella pneumoniae* Infection: Hypermucoviscosity, Virulence Factor, Genotype, and Antimicrobial Susceptibility. Diagnostics.

[19] Li Y.T., Xiao Y.H., Liu Y., Hu N., Wu C., Huang X., Zeng L. (2024). Characterisation of Highly Virulent and Colistin-Resistant ST367-KL1 *Klebsiella quasipneumoniae* subsp. *similipneumoniae* Strain. J. Glob. Antimicrob. Resist..

[20] Sękowska A., Konechnyi Y., Carrazco-Montalvo A. (2025). Whole-Genome Sequencing of *Klebsiella quasipneumoniae* subsp. *similipneumoniae* Isolated from a Patient with Pneumonia. Curr. Microbiol..

[21] Pugh R.N.H., Murray-Lyon I.M., Dawson J.L., Pietroni J.L., Roger W. (1973). Transection Of The Oesophagus For Bleeding Oesophageal Varices. Br. J. Surg..

[22] Chow J.W., Yu V.L. (1999). Combination Antibiotic Therapy versus Monotherapy for Gram-Negative Bacteraemia: A Commentary. Int. J. Antimicrob. Agents.

[23] Clinical and Laboratory Standards Institute (2019). Performance Standards for Antimicrobial Susceptibility Testing; Twenty-Ninth Informational Supplement.

[24] Bortolaia V., Kaas R.S., Ruppe E., Roberts M.C., Schwarz S., Cattoir V., Philippon A., Allesoe R.L., Rebelo A.R., Florensa A.F. (2020). ResFinder 4.0 for Predictions of Phenotypes from Genotypes. J. Antimicrob. Chemother..

[25] Fang C.T., Chuang Y.P., Shun C.T., Chang S.C., Wang J.T. (2004). A Novel Virulence Gene in *Klebsiella pneumoniae* Strains Causing Primary Liver Abscess and Septic Metastatic Complications. J. Exp. Med..

[26] Perlaza-Jiménez L., Wu Q., Torres V.V.L., Zhang X., Li J., Rocker A., Lithgow T., Zhou T., Vijaykrishna D. (2020). Forensic Genomics of a Novel *Klebsiella quasipneumoniae* Type from a Neonatal Intensive Care Unit in China Reveals Patterns of Colonization, Evolution and Epidemiology. Microb. Genom..

[27] Ferreira M.L., Araújo B.F., Cerdeira L.T., Toshio C., Ribas R.M. (2019). Genomic Features of a Clinical ESBL-Producing and Colistin-Resistant Hypermucoviscous *K. quasipneumoniae* subsp. *similipneumoniae* from Brazil. Braz. J. Infect. Dis..

[28] Liu Y., Liu X., Xu H., Zhang X., Liu R., Chen M., Qian J., Zheng B. (2024). Genomic and Phenotypic Characterization of ST2012 Clinical *Klebsiella quasipneumoniae* subsp. *similipneumoniae* Harboring *bla*_NDM−1_ in China. BMC Microbiol..

[29] Brinkac L.M., White R., Souza R.D., Nguyen K., Obaro S.K., Fouts D.E., Bradford P.A. (2019). Emergence of New Delhi Metallo-Lactamase (NDM-5) in *Klebsiella quasipneumoniae* from Neonates in a Nigerian Hospital. mSphere.

[30] Jiang J., Terlecky A.J., Shashkina E., Starolis M.W., Chen L., Kreiswirth B.N. (2026). Genomic Analysis of Community-Associated Multidrug-Resistant *Klebsiella quasipneumoniae* subsp. *similipneumoniae* and the Identification of the ST2059-KL1 Clone in the U.S. Microbiol. Spectr..

[31] Choi M., Hegerle N., Nkeze J., Sen S., Jamindar S., Nasrin S., Sen S., Permala-Booth J., Sinclair J., Tapia M.D. (2020). The Diversity of Lipopolysaccharide (O) and Capsular Polysaccharide (K) Antigens of Invasive *Klebsiella pneumoniae* in a Multi-Country Collection. Front. Microbiol..

[32] Hwang W., Wantuch P.L., Bernshtein B., Zhiteneva J.A., Slater D.M., Vater K.H., Sridhar S., Oliver E., Roach D.J., Rao S.R. (2025). Antibody Responses in *Klebsiella pneumoniae* Bloodstream Infection: A Prospective Cohort Study. Lancet Microbe.

